# Effect of food insecurity on depression, anxiety, and stress among resettled Syrian refugees in Ontario

**DOI:** 10.1371/journal.pgph.0002571

**Published:** 2024-03-15

**Authors:** Safoura Zangiabadi, Baraa Alghalyini, Fatmeh Zoubi, Hala Tamim

**Affiliations:** 1 School of Kinesiology and Health Sciences, York University, Toronto, ON, Canada; 2 Department of Family and Community Medicine, Alfaisal University, Riyadh, Saudi Arabia; 3 Department of Community and Mental Health Nursing, Jordan University of Science and Technology, Irbid, Jordan; York University, CANADA

## Abstract

Food insecurity has been linked to adverse health outcomes, particularly among vulnerable populations such as refugees. The aim of this study was to assess the prevalence of food insecurity and its association with depression, anxiety, and stress among resettled Syrian refugee parents in Ontario. This was a cross-sectional study with a total of 540 Syrian refugee parents who resided in Ontario for an average of four years and had at least one child less than 18 years who were interviewed. Information about food insecurity was collected based on the question “During the past year, did you ever eat less because there was not enough food or money for food?”. Levels of depression, anxiety, and stress were assessed using the Depression Anxiety Stress Scales (DASS-21). Multiple linear regression analyses were performed to evaluate the relationship between food insecurity and depression, anxiety, and stress after adjusting for sociodemographic, migration-, and health-related factors. 44.6% of respondents reported experiencing food insecurity. Of participants, 7.6%, 8.9%, and 8.5% reported severe to extremely severe levels of depression, anxiety, and stress, respectively. Results of the multiple linear regression analysis showed that food insecurity was significantly associated with higher levels of depression (Adjβ = 2.00, p = 0.008), anxiety (Adjβ = 1.53, p = 0.013), and stress (Adjβ = 1.87, p = 0.019). Implementation of effective government interventions and frameworks are essential to reduce food insecurity among resettled Syrian refugees to ultimately improve their mental health outcomes and overall well-being.

## Introduction

Since the start of the Syrian civil war in 2011, more than 14 million people have been forcibly displaced from their home country due to violence, conflict, and persecution [1]. Many countries across the world responded to this humanitarian crisis by resettling these refugees, with Canada as one of the top countries to host Syrian refugees. In response, the Government of Canada had welcomed, from November 2015 to March 2023, over 93,000 Syrian refugees to more than 350 communities across the country [2]. The refugees were resettled through one of the three resettlement programs: Government Assisted Refugees (GARs), Privately Sponsored Refugees (PSRs), and the Blended Visa Office Referred Cases (BVORs) Program [3]. Despite large-scale assistance programs, refugees continue to encounter various challenges throughout their resettlement such as lack of financial resources, language barriers, and inadequate access to healthcare [4–6]. Among the numerous socioeconomic risk factors that refugees face, a crucial challenge that remains prevalent is food insecurity, which places this population at an increased risk of developing adverse health outcomes [7].

The Food and Agricultural Organization of the United Nations defines food security as "when all people, at all times, have physical, social, and economic access to sufficient, safe and nutritious food to meet their dietary needs and food preferences for an active and healthy life" [8]. Food insecurity is the absence of one or more of these conditions and has serious negative repercussions on the health status of refugees and has been linked to increased morbidity and mortality, especially among vulnerable groups such as young children, pregnant women, and older adults [9]. For instance, food insecurity has been shown to affect physical health and contribute to the development of chronic diseases, including diabetes and cardiovascular diseases [10]. According to studies, the key influencers of food insecurity include social determinants of health, including immigration status, income, education, and employment [11]. A recent study of Syrian refugee families resettled in two Canadian cities of Toronto and Saskatoon reported low income as a critical barrier to food security, which was shaped by various factors and included food affordability, accessibility and availability [12]. Additionally, another study conducted in 2020 found that among Syrian refugees resettled in Toronto and Saskatoon, the overall prevalence of food insecurity was 84%, with 31% experiencing severe food insecurity, 41% experiencing moderate food insecurity, and the remaining experiencing marginal food insecurity. This study highlighted that households with an annual income below $40,000 were at a greater risk of food insecurity [13].

Previous studies have highlighted the adverse effects of food insecurity on mental health and well-being with studies showing associations with anxiety disorders, stress, and suicidal ideation among the adult general population [14–16]. For example, in a global analysis of 149 countries, food insecurity was associated with poor mental health among adults in different regions, with the prevalence of food insecurity ranging from 18.3% in East Asia to 76.1% in Sub-Saharan African [17]. Particularly, assessing food insecurity among vulnerable populations, such as refugees, is of utmost importance given its crucial implications for public health and the development of policy frameworks for providing essential food aid. Indeed, the existing literature showed that food insecurity increases the risk of anxiety and depression symptoms among refugees [18–20] with a recent study demonstrating a strong relationship between food insecurity and poor mental health among Syrian refugee mothers in Lebanon [21]. To date, the effect of food insecurity on the mental health of Syrian refugees in Ontario has not been extensively examined. Given the accessibility of publicly funded healthcare services in Canada, which maybe different from other host countries like Lebanon and Jordan [9, 22], it is crucial to investigate the psychological impacts of food insecurity among this population. Thus, this study aims to assess the prevalence of food insecurity and its association with depression, anxiety, and stress among resettled Syrian refugees in the province of Ontario. Findings from this study may assist in establishing health policy frameworks, programs, and services to address food insecurity among such vulnerable population to ensure their health and well-being.

## Methods

This cross-sectional study recruited and interviewed a total of 540 Syrian refugee parents between March 2021 and March 2022. The inclusion criteria necessitated participants to be Syrian refugee parents who reside in Ontario, Canada. Also, participants were required to have at least one child under the age of 18 at the time of interview and have resettled in Canada after 2015. The recruitment and interviews of participants were conducted via telephone interviews to comply with social distancing guidelines due to the COVID-19 pandemic. Participant recruitment was conducted using convenience sampling with the assistance of local service organizations. Furthermore, the survey was administered by Research Assistants who could read, write, and speak Arabic, specifically in the Syrian dialect. All participants provided consent prior to participating in the study and received a $20 honorarium for their participation.

The main outcomes of the study were depression, anxiety, and stress among Syrian refugee parents assessed using the Arabic translated version of the Depression Anxiety Stress Scales (DASS-21) [23]. The DASS-21 is a 21-item self-reported questionnaire consisting of seven items for each subscale of depression, anxiety, and stress. The response scale consists of a 4-point Likert Scale with answers ranging from 0 “Did not apply to me all” to 3 “Applied to me very much, or most of the time” and respondents report the presence of symptoms over the past week. The total score of each subscale is multiplied by 2 to obtain the full-scale score ranging from 0 to 42, with higher scores reflecting greater severity of symptoms. The cut-off scores for depression, anxiety, and stress are categorized respectively into normal (0–9, 0–7, 0–14), mild (10–13, 8–9, 15–18), moderate (14–20, 10–14, 19–25), severe (21–27, 15–19, 26–33) or extremely severe (28+, 20+, 34+) [24].

Information about the main independent variable, food insecurity, was collected based on the question “During the past year, did you ever eat less because there was not enough food or money for food?” with answers categorized into binary measures of “Yes” and “No”. Additionally, several other sociodemographic-, migration-, and health-related factors were collected from the participants. The sociodemographic factors considered for the study included gender (being a mother/father), age, number of children, highest level of education (none-elementary/ secondary high school-diploma/ university), employment status (yes/ no), and perceived language proficiency in English or French measured by the question, “Please rate your current overall ability in English or French, whichever you are more comfortable with”, with the answers ranging from 1 representing “Excellent” to 5 representing “Not at all”. The migration-related characteristics addressed the type of sponsorship involved in the process of becoming a refugee (government-assisted refugee (GAR)/ privately sponsored refugee (PSR)/ and other, and number of years in Canada. Health-related factors included information on self-perceived physical health that was collected on a 5-point Likert scale, with 1 representing “Excellent” ranting and 5 representing a “Poor” rating, smoking (yes/ no), alcohol consumption (yes/ no), and food insecurity (yes/no). Additionally, since participant recruitment took place amid the pandemic, information on the fear of COVID-19 was collected and measured using the Fear of COVID-19 Scale (FCV-19S) [25]. The FCV-19S measures how uncomfortable and afraid participants feel about COVID-19, with higher score demonstrating greater fear of COVID-19. Lastly, to examine the relationship between self-perceived socioeconomic status (SES) and food insecurity, the variable SES was measured by the question “In your current condition, here in Canada, would you say most people would categorize a household like yours as”, with response options ranging from 1 representing “Lower income” to 5 representing “Upper income”.

Simple linear regression models were conducted to assess the bivariate relationship between each of the sociodemographic-, migration-, and health-related factors with satisfaction with stress, anxiety, and depression. Additionally, multiple linear regression analyses were conducted after examining the assumptions and observing no significant violations. Thus, three multiple regression models were performed for the three outcome variables of stress, anxiety, and depression, with the main independent variable of food insecurity as well as other sociodemographic-, migration-, and health-related variables. The beta coefficient and 95% confidence intervals (95% CIs) were reported. All regression models were adjusted for the clustering effect of belonging to the same family. All analyses were conducted using the Statistical Package for the Social Sciences (SPSS, version 28.0).

## Ethics statement

The project was approved by the Research Ethics Board at York University (Certificate # e2019-128) and was conducted in accordance with the ethical standards of the Helsinki Declaration. Informed consent was obtained from all participants. To comply with social distancing recommendations due to the COVID-19 pandemic, questionnaire surveys were conducted remotely over the phone. Before the administration of the questionnaire, the research assistant sent electronically to participants a soft copy of the consent form. The participants were informed about the objectives of the study and the voluntary nature of their participation, with the option to opt-out at any point without any repercussions. Additionally, individuals who decided not to participate received the same treatment and benefits as those who participated. The research assistant then went over the consent form and answered any questions the participants had. The research assistant then recorded the audio of the participants’ oral consent.

## Results

**Fig 1:**
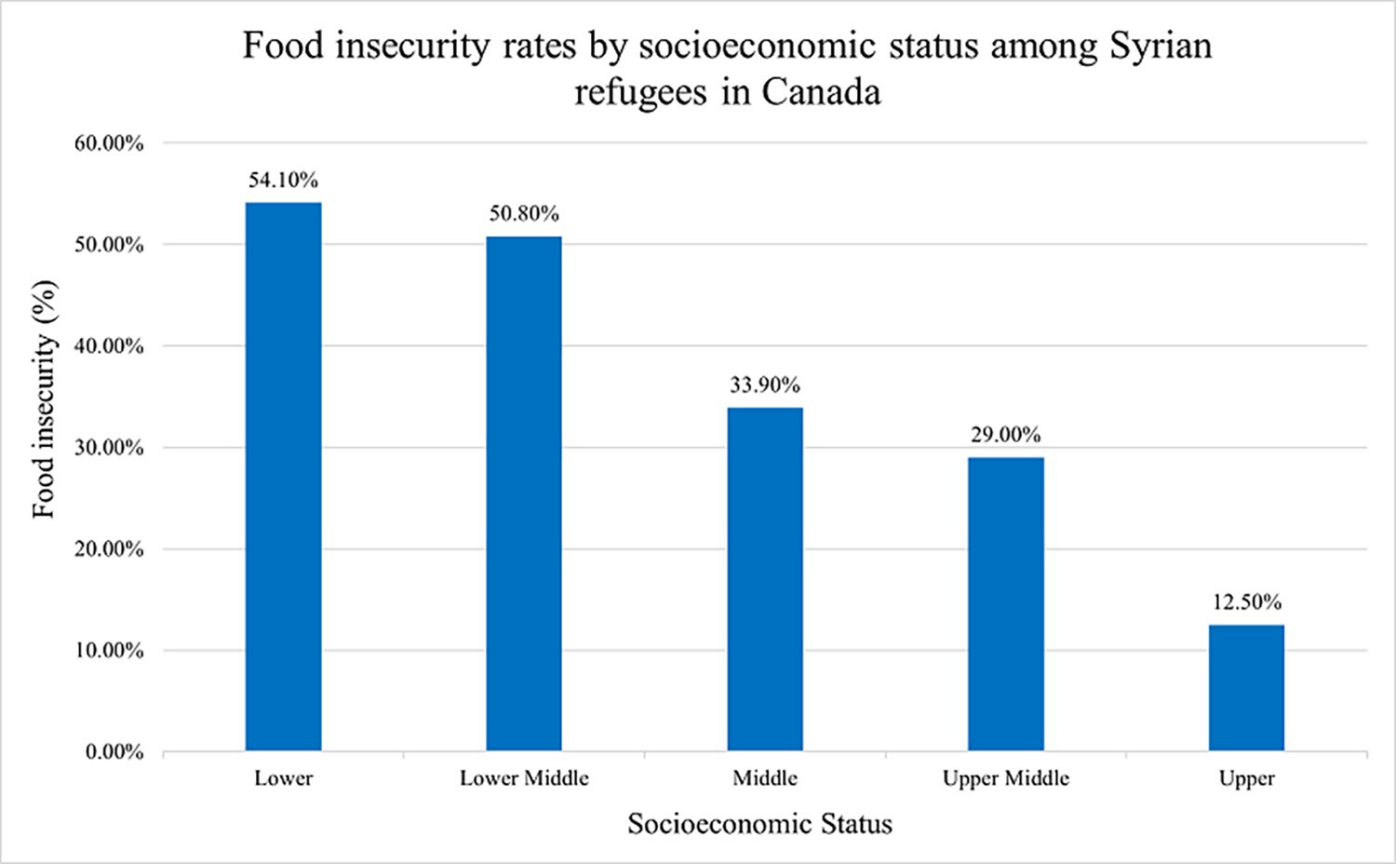
Food insecurity rates by socioeconomic status among Syrian refugees in Canada.

Table 1 presents the descriptive statistics of the study participants’ characteristics and the bivariate association of food insecurity, socio-demographic-, migration-, and health-related factors with mental health outcomes of depression, anxiety, and stress. Among the total of 540 participants recruited for this study, the average age of respondents was 39.8 years (SD = 7.3), with the majority being mothers (60.9%) and a mean number of 3.4 children (SD = 1.5). Participants spent on average 3.90 years (SD = 1.6) in Canada. Also, more than half of the respondents had a secondary or university level education (81.3%), while 65.6% were unemployed. Furthermore, 44.6% of participants reported eating less in the past year due to limited food availability or financial constraints. Fig 1 demonstrates food insecurity rates by socioeconomic status among Syrian refugees in Canada. Findings presented an inverse relationship between food insecurity and socioeconomic status. The results revealed that individuals from lower and lower middle socioeconomic strata reported food insecurity rates surpassing 50%, while those from middle or upper middle strata exhibited comparatively lower rates of food insecurity.

**Table 1:** Characteristics of study participants and the bivariate association between the food insecurity, socio-demographic-, migration-, and health-related factor and metal health-related outcomes including stress, anxiety and depression.

Factors	N (%)	Mean (SD)	Stress		Anxiety		Depression	
			Unadjusted β (SE)	p-value	Unadjusted β (SE)	p-value	Unadjusted β (SE)	p-value
**Food insecurity**								
Yes	241 (44.63)		2.91 (0.85)	< 0.001	2.67 (0.67)	< 0.001	3.14 (0.80)	< 0.001
No	296 (54.81)		Ref		ref		ref	
Socio-demographic								
**Gender**								
Mother	329 (60.93)		ref		ref		ref	
Father	211 (39.07)		-1.89 (0.85)	0.027	-1.24 (0.67)	0.064	-1.25 (0.80)	0.117
**Age**		39.77 (7.33)	.003 (0.058)	0.961	0.04 (0.05)	0.326	0.05 (0.05)	0.365
**Number of Children**		3.35 (1.47)	0.25 (0.29)	0.399	0.41 (0.22)	0.067	0.23 (0.27)	0.394
**Education**								
None/ Elementary	101 (18.70)		ref		ref		ref	
Secondary/High school/ Diploma	278 (51.48)		-2.02 (1.13)	0.075	-2.75 (0.88)	0.002	-2.00 (1.05)	0.059
University	161 (29.81)		-2.19 (1.26)	0.083	-2.92 (.98)	0.003	-2.24 (1.17)	0.056
**Language proficiency** *		3.03 (1.23)	1.09 (0.34)	0.001	1.33 (0.26)	< 0.001	1.17 (0.31)	< 0.001
**Working status**								
Yes	186 (34.44)		-1.34 (0.89)	0.132	-1.28 (0.70)	0.067	-1.78 (0.83)	0.032
No	354 (65.56)		ref		ref		ref	
Migration								
**Sponsorship**								
Governmental	202 (37.41)		ref		ref		ref	
Private	312 (57.78)		-0.54 (0.89)	0.546	-0.55 (0.70)	0.436	-1.56 (0.83)	0.062
Other	26 (4.81)		1.99 (2.05)	0.333	2.63 (1.58)	0.097	0.61 (1.88)	0.745
**Number of years in Canada**		3.90 (1.57)	0.24 (0.28)	0.393	0.32 (0.22)	0.151	0.15 (0.26)	0.558
Health								
**Alcohol drinking**								
Yes	78 (14.44)		-0.68 (1.20)	0.573	-0.69 (0.94)	0.462	-0.14 (1.12)	0.905
No	462 (85.56)		ref		ref		ref	
**Smoking**								
Yes	119 (22.04)		1.51 (1.01)	0.134	2.35 (0.78)	0.003	1.97 (0.94)	0.036
No	421 (77.96)		ref		ref		ref	
**Self-rated Physical Health** **		2.89 (1.08)	3.85 (0.35)	< 0.001	2.90 (0.27)	< 0.001	3.25 (0.33)	< 0.001
**Fear of COVID-19**		15.56 (6.02)	0.36 (0.07)	< 0.001	0.34 (0.05)	< 0.001	0.30 (0.06)	< 0.001

*Language proficiency in English or French

**Scale from 1–5 (1 = Excellent and 5 = Poor)

Table 2 presents depression, anxiety, and stress levels of study participants. The DASS subscales in the study sample indicated high internal consistency, as indicated by Cronbach’s alpha values of 0.894, 0.848, and 0.899 for depression, anxiety, and stress subscales, respectively. Among respondents, the majority reported normal levels of depression (76.3%), anxiety (74.6%), and stress (82.8%), while 7.6%, 8.9%, and 8.5% reported severe or extremely severe levels of depression, anxiety, and stress, respectively. Participants reported an average score (SD) of 6.21 (9.05) for depression, 4.91 (7.58) for anxiety, and 8.30 (9.65) for stress (Table 2). The results of the bivariate regression analysis showed a positive association between food insecurity with depression (Uadjβ = 3.14, p < 0.001), anxiety (Uadjβ = 2.67, p < 0.001), and stress (Uadjβ = 2.91, p < 0.001) (Table 1). Table 3 shows the results of the multiple linear regression models with an overall R square of 0.234, 0.292, and 0.266 for depression, anxiety, and stress, respectively. After adjusting for all other variables, those who were food insecure had significantly higher levels of depression (Adjβ = 2.00, p = 0.008), anxiety (Adjβ = 1.53, p = 0.013), and stress (Adjβ = 1.87, p = 0.019) when compared to food secure individuals. Also, fathers exhibited significantly lower levels of depression (Adjβ = -1.91, p = 0.035), anxiety (Adjβ = -1.91, p = 0.009), and stress (Adjβ = -2.16, p = 0.022) compared to mothers. Meanwhile, participants with poorer language proficiency in English or French reported significantly higher anxiety (Adjβ = 1.14, p<0.001) and depression levels (Adjβ = 0.83, p = 0.040) than those with better language proficiency. Furthermore, results demonstrated a significant association between self-rated physical health and fear of COVID-19 with depression, anxiety, and stress. Those with poorer self-rated physical health reported significantly higher levels of depression (Adjβ = 2.94, p<0.001), anxiety (Adjβ = 2.47, p<0.001), and stress (Adjβ = 3.76, p<0.001). Similarly, respondents with greater fear of COVID-19 experienced significantly higher levels of depression (Adjβ = 0.21, p<0.001), anxiety (Adjβ = 0.23, p<0.001), and stress (Adjβ = 0.22, p<0.001).

**Table 2:** Depression, anxiety, and stress levels among Syrian refugees in Canada.

	Mental Health Outcomes					
	Depression		Anxiety		Stress	
	N %		N %		N %	
**Normal**	412	76.3%	403	74.6%	447	82.8%
**Mild/Moderate**	82	15.4%	87	16.1%	42	7.8%
**Severe/Extremely Severe**	41	7.6%	48	8.9%	46	8.5%
Mean (SD)	6.21 (9.05)		4.91 (7.58)		8.30 (9.65)	

**Table 3:** Results of multivariate regression analyses of the association between the food insecurity, socio-demographic-, migration-, and health-related factors and mental health-related outcomes including stress for the stress, anxiety and depression.

Factors	Stress			Anxiety			Depression		
	Adjusted β (SE)	95% Confidence Interval	p-value	Adjusted β (SE)	95% Confidence Interval	p-value	Adjusted β (SE)	95% Confidence Interval	p-value
**Food insecurity**									
Yes	1.87 (0.79)	0.32, 3.43	**0.019**	1.53 (0.61)	0.33, 2.72	**0.013**	2.00 (0.76)	0.52, 3.48	**0.008**
No	Ref			ref			ref		
Socio-demographic									
**Gender**									
Mother	ref			ref			ref		
Father	-2.16 (0.95)	-4.02, 0.31	**0.022**	-1.91 (0.73)	-3.34, -0.49	**0.009**	-1.91 (0.91)	-3.68, -0.14	**0.035**
**Age**	-0.09 (0.06)	-0.21, 0.03	0.138	-0.04 (0.05)	-0.13, 0.06	0.439	-0.01 (0.06)	-0.12, 0.10	0.891
**Number of Children**	-0.28 (0.33)	-0.92, 0.36	0.383	-0.22 (0.25)	-0.71, 0.27	0.372	-0.58 (0.31)	-1.19, 0.02	0.060
**Education**									
None/ Elementary	ref			ref			ref		
Secondary/High school/ Diploma	-0.67 (1.18)	-2.99, 1.65	0.571	-1.09 (0.91)	-2.87, 0.69	0.227	-0.48 (1.13)	-2.69, 1.73	0.670
University	-0.11 (1.50)	-3.05, 2.83	0.942	-0.23 (1.15)	-2.49, 2.03	0.842	0.35 (1.43)	-2.46, 3.15	0.809
**Language proficiency** *	0.78 (0.42)	-0.05, 1.61	0.067	1. 14 (0.32)	0.50, 1.78	**<0.001**	0.83 (0.40)	0.04, 1.62	**0.040**
**Working status**									
Yes	1.21 (0.92)	-0.61, 3.02	0.192	0.70 (0.71)	-0.69, 2.10	0.323	0.27 (0.88)	-1.47, 2.00	0.762
No	ref			ref			ref		
Migration									
**Sponsorship**									
Governmental	ref			ref			ref		
Private	1.55 (0.98)	-0.38, 3.47	0.114	1.62 (0.75)	0.14, 3.10	**0.032**	-0.23 (0.94)	-2.06, 1.61	0.809
Other	1.74 (1.82)	-1.84, 5.32	0.340	1.98 (1.38)	-0.73, 4.70	0.152	-0.11 (1.71)	-3.47, 3.26	0.951
**Number of years in Canada**	0.26 (0.28)	-0.29, 0.81	0.360	0.53 (0.22)	0.10, 0.95	**0.015**	0.28 (0.27)	-0.25, 0.80	0.299
Health									
**Alcohol drinking**									
Yes	1.45 (1.15)	-0.80, 3.71	0.206	1.09 (0.89)	-0.65, 2.83	0.220	1.97 (1.10)	-0.20, 4.13	0.075
No	ref			ref			ref		
**Smoking**									
Yes	0.10 (1.00)	-1.86, 2.05	0.924	1.39 (0.77)	-0.12, 2.89	0.071	0.91 (0.95)	-0.96, 2.77	0.339
No	ref			ref			ref		
**Self-rated Physical Health** **	3.76 (0.36)	3.04, 4.47	**<0.001**	2.47 (0.28)	1.92, 3.02	**<0.001**	2.94 (0.35)	2.25, 3.62	**<0 .001**
**Fear of COVID-19**	0.22 (0.06)	0.10, 0.35	**<0.001**	0.23 (0.05)	0.13, 0.32	**<0.001**	0.21 (0.06)	0.09, 0.33	**<0.001**

*Language proficiency in English or French

**Scale from 1–5 (1 = Excellent and 5 = Poor)

## Discussion

The present study examined the prevalence of food insecurity and its association with levels of depression, anxiety, and stress among Syrian refugee parents resettled in Ontario. The findings showed a high level of food insecurity (44.6%) among resettled Syrian refugees. Moreover, the results indicated that food insecurity was significantly associated with higher levels of depression, anxiety, and stress. The findings of this study offer valuable insights that may guide the implementation of public health measures and interventions aimed at alleviating food insecurity and ultimately improving the mental health outcomes of Syrian refugees.

Results of this study revealed that 44.6% of Syrian refugees resettled in Ontario experienced food insecurity. These results contrast with a previously conducted study among resettled Syrian refugees in Toronto and Saskatoon, where a substantially higher prevalence of food insecurity (84%) was reported [13]. The difference in the rate of food insecurity could be attributed to variations in the definition of food insecurity utilized in our study compared to the previous one, where food insecurity was measured using categories of marginal, moderate, and severe, each with its distinct definition and subsequently merged [13]. However, our findings were comparable to the prevalence of food insecurity among Syrian refugee families resettled in Lebanon (49%) and Jordan (55%) in 2021 [26, 27]. Indeed, the high prevalence of food insecurity experienced by Syrian refugees resettled in Ontario may be attributed to various factors. For instance, the COVID-19 pandemic disproportionately impacted vulnerable populations, potentially contributing to a higher prevalence of food insecurity within the Syrian refugee population as well [28]. Moreover, the financial hardship resulting from the pandemic exacerbated the pre-existing disparities and socioeconomic inequalities faced by the refugees, preventing them from accessing adequate nutritious food [29]. Additionally, the implementation of social distancing measures and the closure of public spaces due to the pandemic posed considerable challenges for community organizations in their efforts to provide services aimed at alleviating food insecurity [30], possibly exacerbating this issue further. Notably, a comparable trend is also evident among the Canadian general population when considering the impact of the pandemic on food insecurity. According to Canadian Income Survey (CIS) findings, the rate of food insecurity in Ontario increased from 17.1% to 19.2% between 2019 and 2022, reflecting the effect of the pandemic on food insecurity across the broader population [31, 32].

The results of this study showed that 7.6%, 8.9%, and 8.5% of participants had severe or extremely severe symptoms of depression, anxiety and stress, respectively. Similarly, a study of Syrian refugee mothers living in Lebanon revealed that 11.1% and 9.9% of participants suffered from moderate and severe depression, respectively [21]. Another study of Syrian refugees in Turkey showed that the prevalence of depression and anxiety was 36.1% and 34.7%, respectively [33]. The lower prevalence of adverse mental health outcomes among resettled Syrian refugees in Ontario could be possibly attributed to Canada’s provision of free mental health support and access to primary care services, which contributes to improved mental health and well-being of refugees [34]. Also, another likely explanation is that as part of the Syrian resettlement initiative in Canada, refugees are offered services and programs such as language training, help finding suitable and affordable housing, and community support that fosters social integration and perhaps mitigates adverse mental health outcomes [35]. Furthermore, the sense of safety and stability in living circumstances that Syrian refugees experience upon their resettlement in Canada after a long period of exposure to war, violence, and distress may contribute to more favourable mental health outcomes [36].

Findings from this study are consistent with previous literature that highlights the effect of food insecurity on mental health outcomes [18]. A recent study of Syrian refugees resettled in Norway found that severe food insecurity was strongly linked to refugees’ mental health symptoms of anxiety and depression [7]. These findings reflect previous research, which also demonstrated the associations between food insecurity and adverse mental health outcomes even in high-income countries such as Canada and New Zealand [37, 38]. Indeed, previous studies have suggested several pathways that connect food insecurity to mental health. For example, a study by Weaver et al. [39] suggested that the influence of food insecurity on mental health is driven directly by the experience of basic needs deprivation, such as worrying about the source of one’s next meal rather than nutritional needs. Also, the feeling of shame and helplessness associated with financial constraints that hinder access to culturally appropriate foods influences mental health [40]. Likewise, food insecurity appears to compound the existing vulnerabilities of refugees who already experienced traumatic events and stressors associated with displacement, leading to a further decline in their mental health.

Besides, consistent with previous literature, Syrian refugee mothers showed significantly higher levels of depression, anxiety, and stress than fathers [33, 41, 42]. Similarly, a study of newly resettled Syrian refugees in the United States showed that women were almost twice more likely to experience anxiety and depression than men [43]. A possible explanation for this finding may be that females are more exposed to gender-based violence, traumatic events, and at a greater risk of losing their spouse during war and displacement [42, 44]. Moreover, results of the present study indicated that there was no significant association between education level and mental health outcomes. This was in line with a comprehensive systematic review encompassing 15 studies involving Syrian refugees resettled in 10 different countries of which 12 studies reported no significant correlations between the level of education and mental health [45]. However, several studies conducted globally have found that refugees with higher education levels experience poorer mental health outcomes than those with moderate education levels [46–48]. A possible explanation could be that individuals with higher education levels may encounter higher rates of underemployment and thereby greater stress associated with not securing a job that matches their skills and qualifications, ultimately resulting in poorer mental health outcomes [49].

Among health-related factors, self-rated physical health and fear of COVID-19 were significantly associated with mental health outcomes. Indeed, the link between physical health ailments and mental health symptomology has been well established [50–52]. For example, a study of Syrian refugees in Jordan reported that physical activity was associated with a lower likelihood of symptoms of depression, anxiety, and insomnia [50]. Comparably, our findings showed that individuals with poorer self-rated physical health reported higher levels of depression, anxiety, and stress. This could be explained as those with poor physical health may be less likely to participate in activities and engage in social connections. Hence, the isolation and lack of belonging to a social network can result in feelings of loneliness, depression, and anxiety [53]. Furthermore, individuals with greater fear of COVID-19 experienced higher levels of depression, anxiety, and stress. This could be explained as COVID-19 serves as a reminder of the past traumatic events and difficulties that refugees experienced, which contributes increased mental health symptoms [54, 55].

## Limitations

Although this is the first study to assess the influence of food insecurity on the mental health of Syrian refugees resettled in Ontario, several limitations were identified. First, given the cross-sectional nature of the study design, the direction of causality may not be established between the variables. Second, there is a possibility of selection bias as participation was voluntary in the study. Third, there might be information bias where the question concerning food insecurity employed in the study may not comprehensively capture the extent of food insecurity experienced by the participants and since all responses were self-reported by the participants. Fourth, results may be subject to confounding bias, such as medical history of psychiatric or chronic diseases, coping abilities and resilience in the face of difficulties that were not collected in this study. Fifth, while the assessment of food insecurity relied on a single question that aligned with the primary outcome of this study, it may not comprehensively capture the nuanced and complex nature of participants ’ experiences with food insecurity.

## Conclusion

This study investigated the impact of food insecurity on Syrian refugees’ levels of depression, anxiety, and stress in Ontario. Results indicated a high level of food insecurity (44.6%) among resettled refugee parents in Ontario. Moreover, results demonstrated that food insecurity was significantly associated with poor mental health outcomes, including depression, anxiety, and stress. These findings highlight the need to establish policies, programs, and interventions to address the issue of food insecurity. For instance, providing food aid programs to ensure refugees have access to adequate nutrient-rich food. Another strategy would be providing additional job opportunities for resettled refugees and eliminating barriers to employment in order to improve the financial circumstances of households, ensuring that refugees have sufficient funds to meet their basic needs. These measures could contribute to enhanced mental health outcomes among this vulnerable population and promote their well-being.
